# Adapting single-walled carbon nanotube-based thin-film transistors to flexible substrates with electrolyte-gated configurations using a versatile tri-layer polymer dielectric†

**DOI:** 10.1039/d4na01007h

**Published:** 2024-12-23

**Authors:** May Ourabi, Roslyn S. Massey, Ravi Prakash, Benoît H. Lessard

**Affiliations:** a Department of Chemical and Biological Engineering, University of Ottawa 161 Louis Pasteur Ottawa Ontario K1N 6N5 Canada benoit.lessard@uottawa.ca; b Department of Electronics Engineering, Carleton University 1125 Colonel By Drive Ottawa Ontario K1S 5B6 Canada raviprakash@cunet.carleton.ca; c School of Electrical Engineering and Computer Science, University of Ottawa 800 King Edward Ave. Ottawa Ontario K1N 6N5 Canada

## Abstract

Flexibility has been a key selling point in the development of carbon-based electronics and sensors with the promise of further development into wearable devices. Semiconducting single-walled carbon nanotubes (SWNTs) lend themselves well to applications requiring flexibility while achieving high-performance. Our previous work has demonstrated a tri-layer polymer dielectric composed of poly(lactic acid) (PLA), poly(vinyl alcohol) with cellulose nanocrystals (PVAc), and toluene diisocyanate-terminated poly(caprolactone) (TPCL), yielding an environmentally benign and solution-processable n-type thin-film transistor (TFT). Despite the potential for fabrication on flexible substrates, these devices were only characterized on rigid substrates. We present herein the fabrication of these TFTs on Kapton® substrates and a progression of the devices' n- and p-type operation over 7 days, demonstrating continuous loss of the n-type performance and relative stability of the p-type performance after 3 days in ambient air. The tri-layer dielectric is then applied in an electrolyte-gated SWNT field-effect transistor (EG-SWNT-FET) architecture, shielding the SWNTs from the electrolyte and allowing for width-normalised *g*_m_ values of 0.0563 ± 0.0263 μS μm^−1^ and *I*_ON/OFF_ ratios of 10^3^–10^4^ using de-ionized (DI) water as the electrolyte. Finally, as a proof of concept, the device was used to detect α-synuclein, a neuronal protein whose aggregation is associated with Parkinson's disease, in DI water through the immobilization of target specific aptamer molecules on the polymer layer covering the gate electrode.

## Abstract 2

Carbon nanotube-based thin-film and electrolyte-gated transistors with environmentally benign dielectric materials can be fabricated on flexible substrates and have potential for further development into wearable sensors.

## Graphical abstract

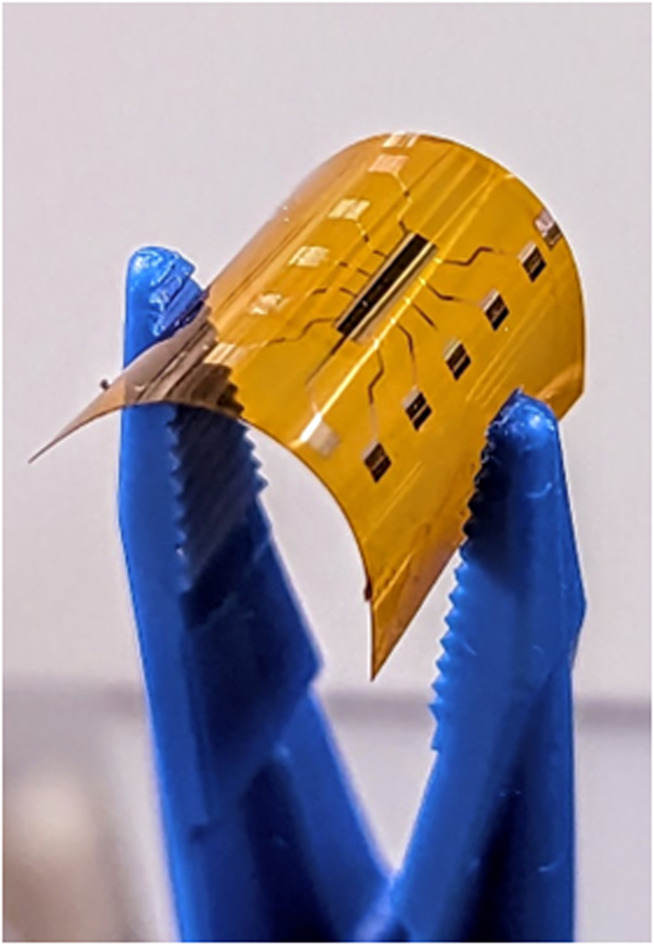

## Introduction

The development of high-performance, flexible thin-film transistors (TFTs) and TFT-based sensors requires high-capacitance dielectric materials and high-mobility semiconductors.^1,2^ Single-walled carbon nanotubes (SWNTs) are a promising semiconducting material due to their ability to withstand mechanical deformation^3,4^ while achieving high source drain currents (*I*_DS_) and transconductance (*g*_m_) values.^5^ When employed in TFTs, the corresponding dielectric layer must be carefully selected to produce high capacitance while also providing the necessary hydrophobic surface to ensure favourable orthogonal processing of the semiconductor^6,7^ and to avoid polar environment at the semiconductor–dielectric interface.^8^

The Lessard group has recently demonstrated that poly(vinyl alcohol) (PVA) is a promising water soluble and biodegradable dielectric^9^ in TFTs and that its hydrophilic/hydroscopic surface can be improved through the coating of a monolayer of hydrophobic poly(caprolactone) (PCL).^10^ We demonstrated that thermal^11^- and UV^12^-crosslinking can be used to couple the PCL to the PVA layer leading to a high-performance bilayer dielectric for the deployment of SWNT-based TFTs. The bilayer crosslinking strategy has enabled the fabrication of high performance SWNT TFTs with *g*_m_ = 159 μS and *I*_ON/OFF_ = 10^5^ which is among the state of the art for random networks of SWNT-based devices. While high-performing, these devices were fabricated on rigid substrates, which is not ideal for the development of wearable technologies.^13,14^

SWNTs typically demonstrate air-sensitive n-type behaviour due to oxygen doping^15^ and sensitivity to moisture.^16,17^ Encapsulation is therefore often required if glovebox operation is inapplicable.^18–20^ However, some applications do not allow for encapsulation of the entire device. For example, when integrating SWNT into biosensors based on electrolyte-gated transistors, the SWNT semiconductor is often exposed directly to an aqueous electrolyte solution as the dielectric material.^21^ While electrolyte-gated transistor sensors are subject to a Debye length limitation, aptamers have been a promising biofunctionalization element due to their small size, which allows for a sensing response in solutions with high ionic strength.^22–24^

SWNT-based aptasensors have been demonstrated both on rigid^25–28^ and flexible substrates,^29–31^ although these are conventionally fabricated by immobilizing the aptamer onto the SWNT. Analyte detection is therefore linked to the electrostatic gating effect induced by the binding events taking place at the surface of the nanotube.^29^ However, direct immobilization of an aptamer onto the nanotube would lead to defects, while non-covalent bonding using linker molecules^32,33^ has not been extensively studied when paired with polymer-wrapped SWNTs, which represent a growing share of SWNTs used in the literature due to their ease of separation and scalability.^34,35^ Moreover, traditional electrolyte-gated devices wherein the semiconductor is in direct contact with the electrolyte suffer from high leakage currents.^36^ Although these can be mitigated with the addition of an electrode encapsulation layer, this additional fabrication step requires high accuracy to ensure only the source and drain electrodes are covered.^37^

The Prakash group has developed an alternative to the conventional electrolyte gate aptasensor architecture by physically isolating the semiconductor and gate electrode from the electrolyte using thin polymer layers.^38–40^ The aptamers can then be immobilized onto the polymer layer covering the gate. To improve the performance of the previously reported organic electrolyte-gated field-effect transistors (OEGFET), we fabricated devices with semiconducting SWNTs paired with the tri-layer dielectric on polyimide (Kapton®) substrates. This first required the fabrication of TFTs on Kapton® to evaluate the change in performance when substituting the rigid, ultra-flat substrate for a flexible substrate. We then report the fabrication of electrolyte-gated SWNT field-effect transistors (EG-SWNT-FET) analogous to the OEGFETs and present a proof of concept wherein the device is used as an aptasensor for the detection of α-synuclein.

## Experimental

### Materials

Poly(lactic acid) (PLA, *M*_n_ = 40 000 Da) and poly(vinyl alcohol) (PVA, *M*_w_ = 31 000–50 000 Da) were purchased from Sigma-Aldrich. Toluene diisocyanate-terminated poly(caprolactone) (TPCL) was synthesized using previously a reported procedure^41^ from poly(caprolactone) (PCL) diol (*M*_w_ = 2000 Da) and toluene-2,4-diisocyanate (TDI, 98%), which were also purchased from Sigma-Aldrich. Cellulose nanocrystals (CNCs) were donated by FPInnovations in a 2 wt% aqueous dispersion. 25 μm-thick flexible polyimide sheets (Kapton®) were purchased from DuPont de Nemours, Inc.

For the fabrication of EG-SWNT-FET devices, 950A4 poly(methyl methacrylate) (PMMA, *M*_n_ = 950 000 Da), PCL (*M*_n_ = 48 000–90 000 Da), PVA (*M*_n_ = 30 000–70 000 Da), and poly(3,4-ethylenedioxythiophene) polystyrene sulfonate (PEDOT-PSS) were purchased from Sigma-Aldrich. The α-synuclein monomer was purchased from rPeptide, and the α-synuclein monomer-specific aptamer was synthesized by the Laboratory for Aptamer Discovery and Development of Emerging Research (LADDER) at Carleton University.^42^

### Conjugated polymer sorting of SWNTs

Raw plasma torch single-walled carbon nanotubes (SWNTs) were purchased from Raymor NanoIntegris (batch #RNB781-120). The SWNTs were dispersed with poly(9,9′-didodecylfluorene-*co-N*-(2′-decyltetradecane)-carbazole) (PCPF) (*M*_n_ = 65, PD = 2.7) in toluene and purified following procedures previously reported in literature.^43^ Briefly, a 0.8 : 1 mixture of polymer to raw SWNTs was added to 20 mL of toluene and sonicated for 90 minutes in a chilled bath sonicator (VWR 2.8 L Ultrasonic Bath Cleaner). The dispersion was then centrifuged at 15 000*g* for 15 to 20 minutes while maintaining a temperature of 10 °C (Thermo Scientific Sorvall ST 16R, Fiberlite F15 Fixed Angle Rotor). UV-vis-NIR (Agilent Technologies Cary 7000) and Raman spectroscopy (Renishaw inVia Confocal Raman microscope) were used to assess the purity of the semiconducting SWNTs. If needed, an additional treatment with silica gel was used to remove trace amounts of metallic SWNTs.^44^ The concentration of the dispersion was adjusted by concentrating the dispersion until the S_22_ peak found at 940 nm in the UV-vis-NIR spectrum reached an absorbance of 2.0 a.u. The excess polymer was kept in the dispersion.

### TFT device fabrication

Kapton® sheets were sequentially rinsed with acetone, isopropyl alcohol (IPA) and deionized (DI) water. The surfaces were then dried under nitrogen flow and treated with air plasma for 15 minutes (Harrick Plasma Cleaner, model no. PDC-32 G), after which they were rinsed with DI water and IPA. Once dry, the Kapton® was submerged in a 1% v/v solution of octyltrichlorosilane (OTS) (Sigma-Aldrich) in toluene for one hour at 70 °C, rinsed with toluene and dried under vacuum at 70 °C for one hour. Source and drain electrodes composed of 2 nm of Cr and 50 nm of Au were patterned onto the substrates *via* physical vapour deposition (PVD) (Angstrom Engineering EvoVac Thermal Evaporator) through custom shadow masks. Each substrate yielded 5 sets of source and drain electrodes with *W* = 400 μm and *L* = 50 μm.

The SWNT networks were cast by first transferring approximately 1 mL of the dispersion to a syringe (Hamilton 10 mL Gastight Syringe Model 1010 TLL), which was then affixed to a desktop robot (Musashi SHOT Mini 200Sx) fitted with an automated drop-dispenser (Nano Master SMP-III). The desktop robot was programmed to place 0.2 μL drops on the channel locations. The substrates were then rinsed with toluene at a 45° angle and dried with nitrogen four times. This was followed by an annealing process at 200 °C under vacuum for 1 hour.

The tri-layer dielectric was deposited by spin-coating each polymer layer sequentially (Laurell WS-650-23 B Spin Coater) for 90 seconds at 1500 rpm, as previously reported.^10^ First, a 2 mg mL^−1^ solution of PLA in chloroform was coated statically, immediately followed by an 80 mg mL^−1^ solution with PVA with a 0.75 wt% loading of cellulose nanocrystals. The obtained bilayer was annealed under vacuum at 150 °C for one hour. A 2 mg mL^−1^ solution of TPCL in toluene was then statically spin-coated onto the substrates and annealed at 200 °C for 15 minutes under vacuum. A 50 nm layer of Au was patterned over the device channels to form the gate electrode, yielding a top-gate bottom-contact (TGBC) architecture. Prior to testing, the polymer layer covering the source and drain electrode contact pads was removed using a blade.

### EG-SWNT-FET device fabrication

In both EG-SWNT-FET architectures, the bottom layer was fabricated similarly to the TFTs, excluding the gate electrode patterning step. In contrast to the TFTs, the source-drain electrodes were composed of 50 nm Cr and 50 nm Au instead of 2 nm Cr and 50 nm Au. In the first EG-SWNT-FET architecture, the top layer of the device was fabricated on a separate Kapton® sheet. The gate electrode was first patterned by PVD (Angstrom Engineering Covap System) through a shadow mask using a 50 nm layer of Al and a 50 nm layer of Cr. A 200 nm layer of PMMA was then spin-coated statically at 4000 rpm for 45 seconds. The microchannel was then formed by affixing moulded PCL strips to the PMMA layer using a solution of 5% PCL in chloroform as an adhesive. The aptamers were immobilized onto the PMMA surface by first drop casting 10 μL of a 1 mg mL^−1^ solution of α-synuclein aptamer into the microchannel area, followed by UV activation (180 nm wavelength) for 7 minutes. The surface was then dried in a nitrogen atmosphere for 1 hour.

In the second EG-SWNT-FET architecture, the microchannel and the second layer of the device fabricated by casting a PCL in a mould to produce a microchannel with a height of a 300 μm. The gate electrode was extrusion printed using PEDOT:PSS (3D Bioplotter, EnvisionTEC). Copper sheeting was then added to ensure efficient gate contact.

### TFT device testing

Devices were tested in air under ambient conditions immediately after removal from a nitrogen environment, and subsequently stored in air away from light. A Keithley 2614B sourcemeter was used in conjunction with a custom LabView program to collect output and transfer curves in p- and n-type operation. To collect output characteristics, the source-gate voltage (*V*_GS_) was varied between 0 V and ± 5 V while sweeping the source-drain voltage (*V*_DS_) from 0 V to ± 5 V. Transfer characteristics were collected in the linear regime by applying a *V*_DS_ of ± 1 V and sweeping *V*_GS_ bidirectionally from ± 5 V to ∓ 5 V on the first day, and from ± 5 V to ∓ 7 V when testing on later dates. The transconductance (*g*_m_) was taken as the slope of the transfer curve:
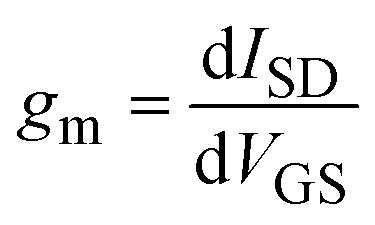


The threshold voltage was also calculated in the linear regime, as shown below.
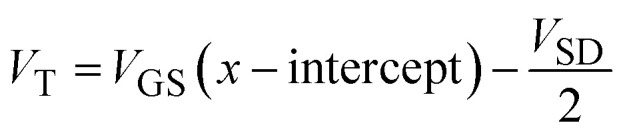


When collecting transfer characteristics, *V*_GS_ was applied at a 1 Hz frequency by pulsing on for 200 ms and off for 800 ms at each data point.

### EG-SWNT-FET device testing

Devices were tested immediately post-assembly under ambient conditions using an HP 4156 Semiconductor Parameter Analyzer. Output curves were collected by varying *V*_GS_ between 0 V and −1.4 V and sweeping *V*_DS_ from 0 V to −1 V. Transfer curves were collected in the linear regime at *V*_DS_ = −0.125 V by sweeping *V*_GS_ from 0.2 V to −1 V. The microchannel was filled with DI water and 1× TAE buffer solution to determine the device characteristics. When testing the EG-SWNT-FET as a sensor, the microchannel was filled with various dilutions of α-synuclein in DI water and rinsed with DI water between tests.

## Results and discussion

### Fabrication of thin-film transistors on Kapton® substrates

High-performance SWNT devices on Kapton® substrates were fabricated using a previously reported tri-layer polymer dielectric consisting of poly(lactic acid) (PLA), poly(vinyl alcohol) (PVA) blended with cellulose nanocrystals (CNCs) and toluene diisocyanate-terminated poly(caprolactone) (TPCL).^10^ This combination of polymer dielectric layers leads to improved film formation, decreases the device's sensitivity to ambient humidity levels, eliminates charge traps and decreases leakage current. However, to date this promising dielectric has ony been applied to rigid glass and silicon wafers. Polyimide (Kapton®) substrates were employed due to their superior chemical and thermal resistance. Substrates were treated with octyltrichlorosilane (OTS) to increase the hydrophobicity of the surface, resulting in greater SWNT surface coverage which is known to lead to improved TFT performance.^45^ Water contact angle was used to determine whether the treatment was successful, as shown in Table S1.† Drop-dispensing was used as the SWNT deposition technique due to its versatility and simplicity, although the flexible nature of the substrates caused the surfaces to buckle when depositing SWNTs and polymer layers. This may have led to imperfect film formation and did not allow for the collection of accurate profilometry and atomic force microscopy measurements.

**Fig. 1:**
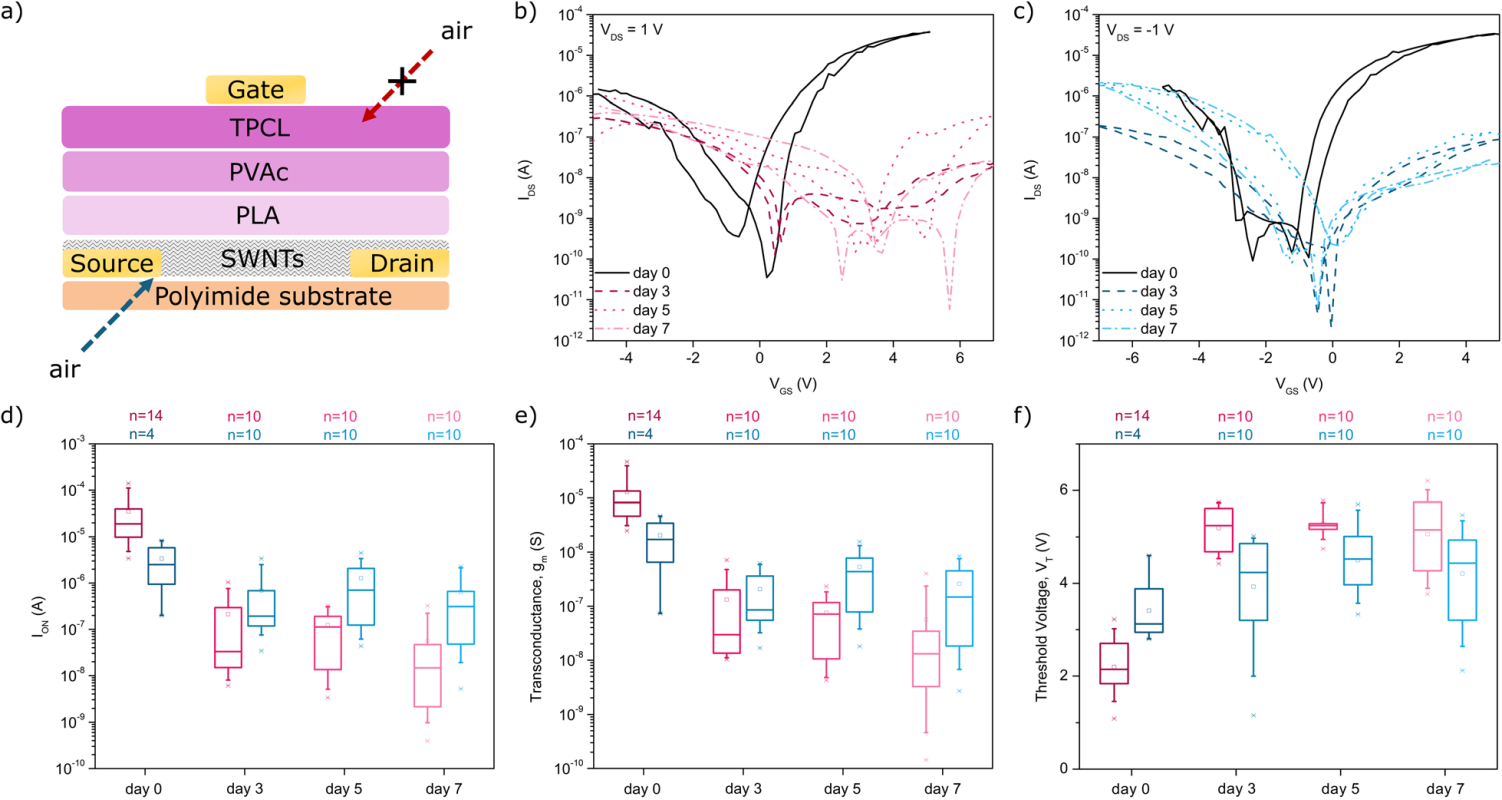
7-Day longevity of the n- and p-type performance of the TFTs fabricated on Kapton®. (a) Schematic of the TFT device architecture, where the Kapton® polyimide substrate is permeable to oxygen. (b) n- and (c) p-type transfer curves obtained from a TFT immediately after exposure to ambient air, and 3 days, 5 days, and 7 days post-exposure. (d) *I*_ON_, (e) *g*_m_, and (f) absolute *V*_T_ obtained from n- and p-type testing over 7 days in ambient air. Data shown in shades of red or blue are indicative of n- and p-type operation, respectively. *n* = 10 devices, with the exception of day 0, where *n*_n-type_ = 14 devices and *n*_p-type_ = 4 devices.

We previously reported the mostly n-type operation of the tri-layer SWNT TFTs on rigid quartz-coated glass substrates.^10^ While some p-type behaviour had been observed immediately post-fabrication, it remained suppressed during the lifespan of the device due to the SWNTs being located between the quartz substrate and the tri-layer polymer, reducing its exposure to oxygen and moisture. Tri-layer-dielectric transistors fabricated on Kapton® substrates similarly exhibit greater n-type performance immediately post-exposure to ambient air, but this rapidly decreases in favour of p-type behaviour due to the permeability of the substrate to oxygen and the resulting gradual exposure of the SWNTs to ambient conditions. This reduction in n-type operation and increase in p-type operation is illustrated in Fig. 1, whereas Fig. S1† displays the change in n- and p-type output characteristics of a device fabricated on Kapton® from its removal from a nitrogen environment to after 3, 5, and 7 days in ambient air.

The day 0 n-type performance of the TFTs on Kapton® demonstrated an average transconductance (*g*_m_) of 13.0 ± 13.1 μS, and a median *g*_m_ of 8.1 μS, which translated to width-normalized average and median transconductance values of 0.032 μS μm^−1^ and 0.020 μS μm^−1^, respectively. In contrast, our previously reported devices on quartz achieved average transconductance values one order of magnitude greater than those reported herein and did not display comparable variability in the results. This may be explained by the greater surface roughness of the Kapton®, especially following plasma treatment, leading to more uneven films.^46^ The average and median threshold voltage (*V*_T_) values of the TFTs fabricated on Kapton® were 2.2 ± 0.6 V and 2.1 V, which was also greater than those reported on quartz, while the *I*_ON/OFF_ ratio decreased by an order of magnitude to 10^4^, in agreement with the lowered *I*_ON_ values when fabricating devices on Kapton® substrates. Similar drops in performances have been reported for SWNTs when moving from the use of rigid to flexible substrates.^47^

Most functional n-type devices did not function as p-type devices immediately after their removal from an inert milieu. Only 4 of the 14 devices demonstrated fair p-type behaviour, which is likely due to them having been exposed while other devices were being tested. These p-type devices nonetheless achieved median *g*_m_ and *I*_ON_ values of 1.7 μS and 2.5 μA, one order of magnitude below the median *I*_ON_ of the n-type devices.

Ultimately, 10 of the 14 devices that were initially tested were still functional after having been stored in air away from light for 3 days. To obtain accurate values of *V*_T_, *V*_GS_ was increased to ±7 V. Fig. 1b and c show a comparison of n- and p-type transfer curves from one of these devices. As shown in Fig. 1d–f, the n-type performance was markedly worse than it had been during initial testing, with median relative *g*_m_ and *I*_ON_ values, expressed as the ratio between the value on day 3 and the initial value, on the order of 10^−3^. Meanwhile, all 10 of the devices functioned as p-type devices, with median *g*_m_ and *I*_ON_ values of 85.1 nS and 193 nA. From the third day onward, the median relative *g*_m_ and *I*_ON_ of the n-type devices each decreased by one order of magnitude every two days relative to the previous test. P-type devices, on the other hand, exhibited a slight increase in *g*_m_ and *I*_ON_ from day 3 to day 5, but decreased by an order of magnitude by day 7. A similar trend is observed in the value of absolute *V*_T_.

Hysteresis in the transfer curves, defined as the difference between the value of *V*_T_ calculated from the forward and reverse sweeps, can also be seen to increase from the third day of exposure onwards in Fig. S2.† The results obtained from our longevity study of the TFTs on Kapton® substrates informed our decisions when testing the devices with an electrolyte-gated single-walled carbon nanotube field-effect transistor (EG-SWNT-FET) architecture. All testing was performed in p-type operation no more than one day after exposure to ambient air. This would reduce variability in the results and maintain an accurate comparison between the previously reported EG-SWNT-FETs, which were also tested as p-type devices.

### EG-SWNT-FET device architecture

**Fig. 2:**
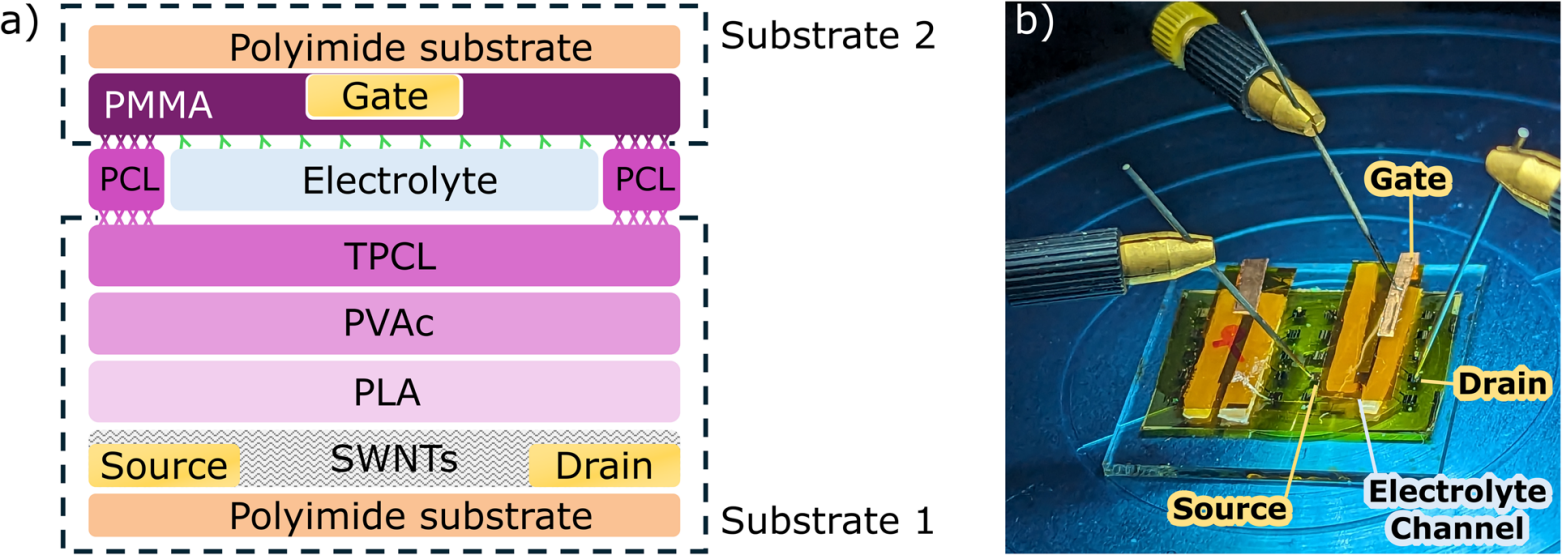
(a) Schematic and (b) photograph of the PMMA-microchannel EG-SWNT-FET device architecture. Elements immobilized onto the polymer layer atop the gate correspond to aptamers.

Electrolyte-gated devices were fabricated using a novel PCL-PMMA microchannel architecture, shown in Fig. 2a. A first Kapton® sheet was used to construct the bottom layer of the device, corresponding to the TFT structure without the gate electrode. The top layer was fabricated by spin-coating a PMMA layer atop a patterned gate electrode on a separate Kapton® sheet. The PCL microchannels shown in Fig. 2b are created by chemically adhering the PCL strips to the PMMA-coated Kapton® surface using a thin layer of 5% PCL in chloroform. The assembly results in a sealed microfluidic channel that spans over an array of 5 devices on the substrate. Similarly, a novel alternative to this configuration was also explored in this study, in which a lone Kapton® substrate is used to fabricate the device and the PCL microchannel is molded out of a single layer and affixed to the tri-layer dielectric. This is shown in Fig. S3.† In both cases, the cavity is then filled with the electrolyte using a syringe, forming the electrolyte channel. The PMMA and PCL layers in contact with the gate electrode and electrolyte medium can also be functionalized with biorecognition elements such as aptamers or antibodies to impart biosensing abilities.

**Fig. 3:**
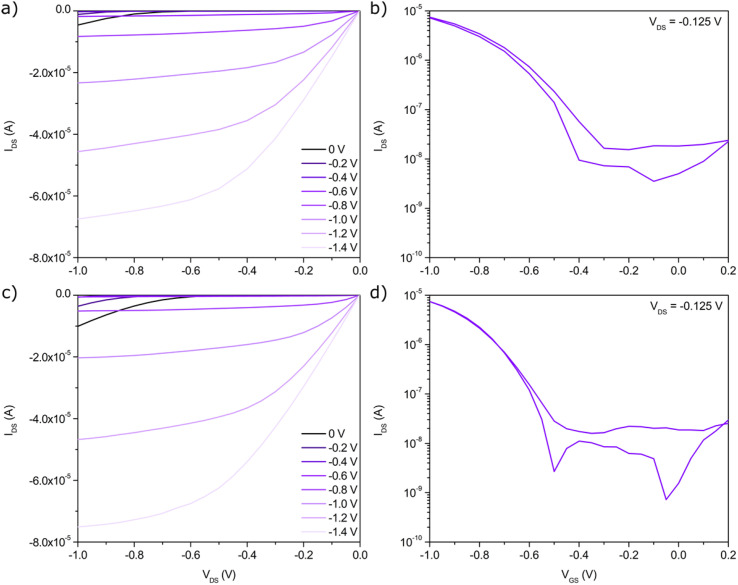
PMMA-microchannel EG-SWNT-FET output and transfer characteristics using (a), (b) DI water and (c), (d) 1× TAE buffer as the electrolyte.

Devices fabricated using the PCL-PMMA microchannel configuration displayed average and median transconductance values of 22.5 ± 10.5 μS and 19.1 μS, translating to width-normalized average and median transconductance values of 0.0563 ± 0.0263 μS μm^−1^ and 0.0478 μS μm^−1^ when using DI water as the electrolyte. These devices also achieved average and median threshold voltages of −0.61 ± 0.06 V and −0.56 V, with *I*_ON/OFF_ ratios of 10^3^–10^4^. Importantly, the currents achieved by the EG-SWNT-FET are comparable to those obtained from the previously reported devices using TIPS-pentacene at much higher voltages, allowing for significantly reduced operating voltages in the EG-SWNT-FET configuration. The devices demonstrated similarly good performance with 1× TAE buffer as the electrolyte, with increased average *g*_m_ values of 29.1 ± 4.2 μS, width-normalized *g*_m_ values of 0.0726 ± 0.0104 μS μm^−1^ and similar *V*_T_ values of −0.63 ± 0.01 V. Illustrative output and transfer characteristics of the devices using both electrolytes are shown in Fig. 3.

The second EG-SWNT-FET architecture, which was fabricated atop a single Kapton® substrate, did not achieve similarly high currents despite increasing *V*_GS_ up to 3.5 V. Although employing 1× TAE buffer as the electrolyte resulted in increased currents, continued operation of the device lead to an increase in leakage currents, as seen in the output curves in Fig. S4.† Ultimately, visual examination of the printed-gate/PCL microchannel revealed that the device succumbed to damage following operation, and this architecture was not retained as a viable platform for the aptasensor fabrication. Output and transfer characteristics of the non-aptamer functionalized PMMA-microchannel EG-SWNT-FET are shown in Fig. S5.†

### Sensor performance

**Fig. 4:**
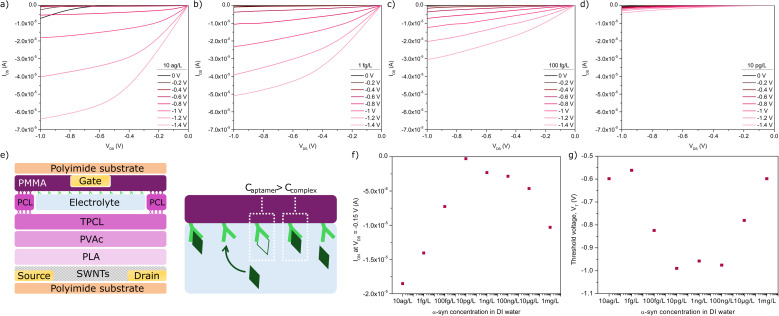
Output characteristics of the EG-SWNT-FET sensor obtained using an electrolyte consisting of (a) 10 ag L^−1^, (b) 1 fg L^−1^, (c) 100 fg L^−1^, and (d) 10 pg L^−1^ α-synuclein in DI water. (e) Schematic of the EG-SWNT-FET demonstrating the process of α-synuclein binding to the α-synuclein-specific aptamer. (f) *I*_ON_ (*V*_GS_ = −1.4 V) of the EG-SWNT-FET in the linear regime (*V*_DS_ = −0.15 V) at all α-synuclein dilutions in DI water. (g) Threshold voltage (*V*_T_) calculated from the linear regime at all α-synuclein dilutions in DI water.

The biosensing ability of the EG-SWNT-FET device was demonstrated using the proof of concept aptasensor architecture using varying concentrations of α-synuclein in DI water between 10 ag L^−1^ and 1 mg L^−1^. The device channel was rinsed with DI water to remove bound and unbound α-synuclein between injections of other dilutions. Despite negligible shifts in threshold voltage at concentrations of 10 ag L^−1^ and 1 fg L^−1^, a noticeable decrease in current is observed from concentrations of 10 ag L^−1^ to 10 pg L^−1^. This change is visible in the output characteristics obtained from the devices, shown in Fig. 4a–d, while Fig. S6† displays the change in output characteristics of the device over the full range of concentrations. The minimum current achieved by the device was observed at a concentration of 10 pg L^−1^. From this concentration up to 100 ng L^−1^, the *V*_T_ ceases to vary while *I*_ON_ increases slightly. Beyond 100 ng L^−1^, the effect of increasing concentration is reversed vis-à-vis the response observed below 10 pg L^−1^, suggesting a possible change in the effective gate capacitance term driving device performance. This can be observed in Fig. 4f and g, as well as in the transfer characteristics and the trend of *g*_m_ with increasing concentration, shown in Fig. S7.† As a negligible proportion of remaining aptamers are left unbound with increasing concentration, excess α-synuclein remains in the electrolyte bulk leading to a more pronounced matrix effect. This also increases the ionic strength of the electrolyte due to the charge inherent to the unbound excess α-synuclein, which provides context for the increase in *I*_ON._ Similar observation were reported for electrolyte-gated field-effect transistor devices using Tips-Pentacene as an organic semiconductor where the presence of an inflection point in the output current of the device,^40^ which corresponds to the transition from a binding effect (sensing region) to a matrix effect where the device is no longer sensitive to the target analyte variation. Ricci *et al.* report an electrolyte-gated FET-based sensor aimed at detecting α-synuclein with a linear range of 0.25 pM to 25 nM, though it made use of antibodies as the recognition element.^48^ Other aptamer-based sensors achieve lower linear ranges, which more closely resemble our range of concentrations, such as photoelectrochemical and electrochemiluminescent sensing platforms,^49,50^ though these examples detect α-synuclein oligomers. Overall, our results demonstrate a proof of concept of how PLA/PVAc/PCL trilayers can be used in a Kapton-substrate EG-SWNT-FET sensor for the detection of α-synuclein.

## Conclusion

This study presents an example of adapting a SWNT-TFT using an environmentally benign tri-layer dielectric to flexible substrates. The impermeability of the dielectric layer to oxygen and moisture afforded the TFTs functional n-type performance without modification to the SWNTs. However, the transition from ultra-flat quartz substrates to Kapton® caused the n-type performance of the TFT to worsen, with observed *g*_m_ and *I*_ON_ values decreasing by an order of magnitude and *V*_T_ shifting by 2 V. This can be related to the increased surface roughness of the substrate. The n- and p-type performance was also observed to shift over 7 days, with p-type performance progressively becoming more dominant due to the exposure of the SWNTs to oxygen *via* diffusion through the Kapton® substrate. After a week, the devices were still functional as p-type devices with median *I*_ON_ and *g*_m_ values having decreased against the baseline n-type performance by one and two orders of magnitude, respectively.

Upon successful fabrication of the SWNT-TFTs on the flexible Kapton® substrate, the device structure was incorporated into an electrolyte-gated architecture, yielding increased *g*_m_ values in sub-volt operation. As a proof of concept, the PMMA-microchannel EG-SWNT-FET was used as an aptasensor for the detection of α-synuclein *via* functionalization of the PMMA layer. The *I*_ON_ and *V*_T_ values were shown to worsen with increasing concentrations of α-synuclein in DI water up to a peak of 10 pg L^−1^.

The currents, *I*_ON/OFF_ ratio and *V*_T_ achieved by the devices reported herein exceed those produced by analogous small molecule organic semiconductor OEGFET devices while maintaining lower operating voltages. Incorporating SWNTs in the sensor architecture further simplifies device processing by reducing reliance on aggressive organic solvents, whilst offering new exploratory approach to incorporating bio-recognition molecules in ordered orientation to further improve sensitivity and detection limit. Moreover, the use of the tri-layer dielectric allows for more environmentally benign components to be integrated into the OEGFET device architecture. The adaptability of the device architecture and the ease with which multiple devices can be fabricated within the same channel allows for potential multiplexing of an array of sensors. This offers promising avenues for the fabrication of flexible, low-power, and low-cost biosensors with improved performance and detection ranges, although extensive testing of the EG-SWNT-FET platform is still required to establish common sensing figures of merit, such as LOD and LOQ. Future work should investigate expanding the bio-recognition molecule footprint to antibodies and nanobodies, and feature the detection of analytes in complex biological specimen, while the EG-SWNT-FET architecture should be extended to a continuously flowing microfluidic channel to expedite testing and ensure liquid samples do not leak out of the device.

## Data availability

The data supporting this article have been included as part of the ESI.†

## Conflicts of interest

There are no conflicts to declare.

## Supplementary Material

NA-007-D4NA01007H-s001
