# Supplementary material for: Adapting single-walled carbon nanotube-based thin-film transistors to flexible substrates with electrolyte-gated configurations using a versatile tri-layer polymer dielectric
Source: Nanoscale Adv. 2024 Dec 23;7(4):1154–62. doi: 10.1039/d4na01007h (PMC11701725; doi:10.1039/d4na01007h)
Supplement: NA-007-D4NA01007H-s001 [file NA-007-D4NA01007H-s001.pdf]

Supplementary Information (SI) for Nanoscale Advances.
This journal is © The Royal Society of Chemistry 2024

Adapting Single-Walled Carbon Nanotube-Based Thin-Film Transistors to Flexible Substrates with Electrolyte-Gated Configurations Using a Versatile Tri-Layer Polymer Dielectric

May Ourabi[1], Roslyn S. Massey[2], Ravi Prakash[2,] and Benoît H. Lessard[1,3,*]*

1: Department of Chemical and Biological Engineering, University of Ottawa, 161 Louis Pasteur, Ottawa, ON, Canada

2: Department of Electronics Engineering, Carleton University, 1125 Colonel By Drive, Ottawa, Ontario K1S5B6, Canada

3: School of Electrical Engineering and Computer Science, University of Ottawa, 800 King Edward Ave., Ottawa, ON, Canada

*corresponding authors: benoit.lessard@uottawa.ca (B.H.L) and raviprakash@cunet.carleton.ca (R.P.)

Supplementary Information

Table S1. Comparison of water contact angle (°) before and after treatment with octyltrichlorosilane (OTS)

	Before OTS treatment	After OTS treatment
Kapton	73.1 ± 1.6°	98.5 ± 1.5°

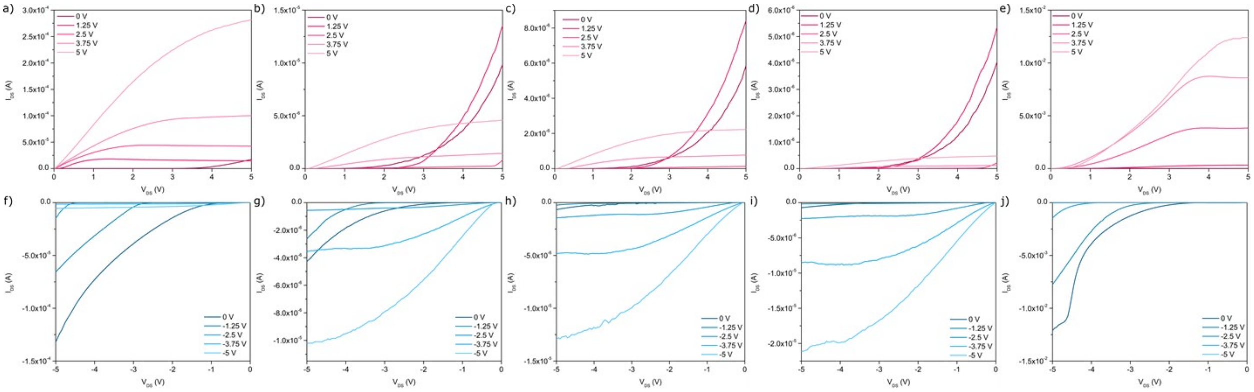

Figure S1. n- (red) and p-type (blue) output characteristics of a tri-layer polymer dielectric TFT fabricated on Kapton® after (a, f) 0 days, (b, g) 3 days, (c, h) 5 days, and (d, i) 7 days exposure to ambient air. (e) n- and (j) p-type output characteristics of a tri-layer polymer dielectric TFT fabricated on quartz after 7 days exposure to ambient air.

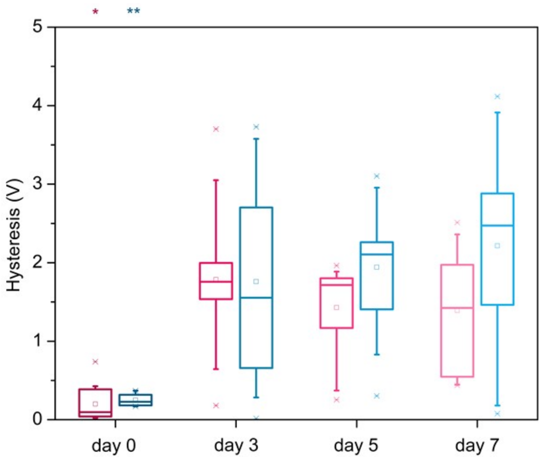

Figure S2. Hysteresis obtained from n- and p-type testing over 7 days in ambient air.

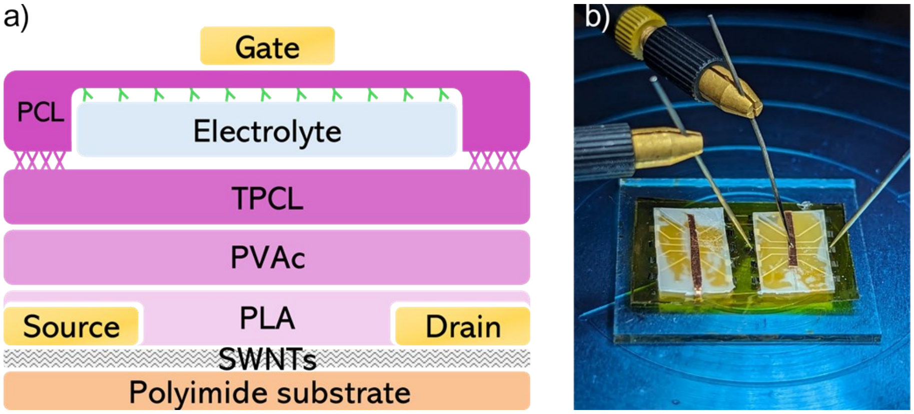

Figure S3. a) Schematic and b) photograph of the PCL-microchannel EG-SWNT-FET device architecture.

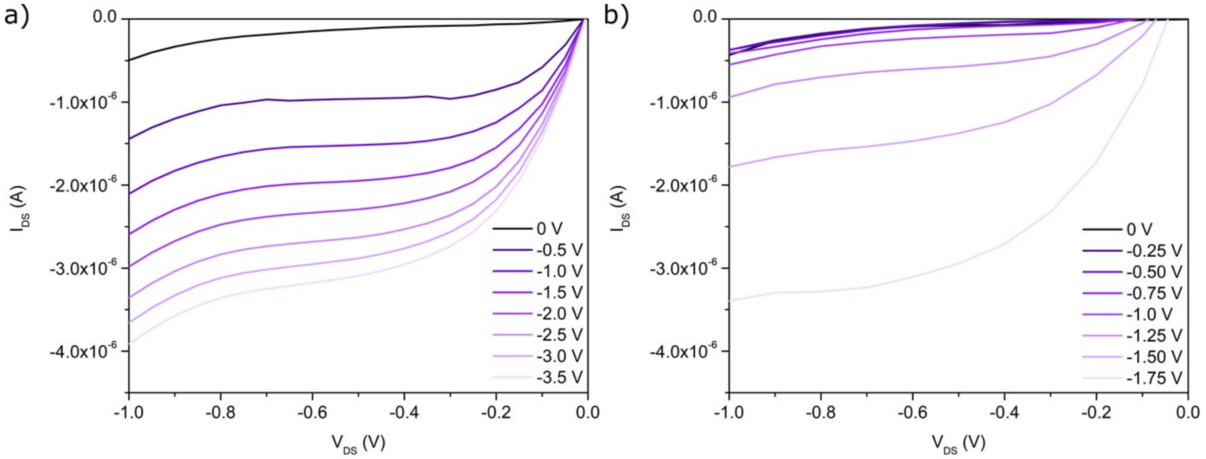

Figure S4. PCL-microchannel EG-SWNT-FET output characteristics using a) DI water and b) 1X TAE buffer as the electrolyte.

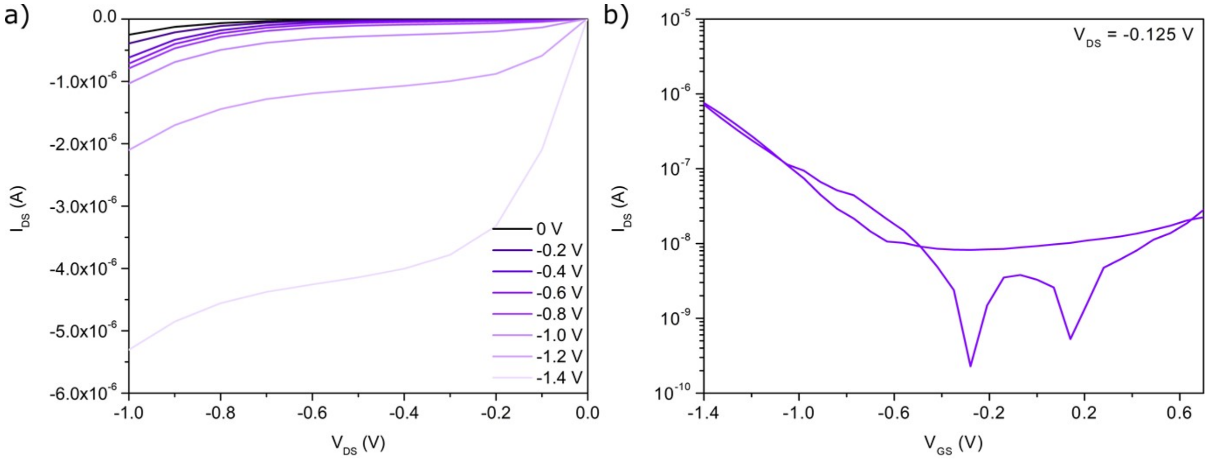

Figure S5. Non-α-synuclein specific PMMA-microchannel EG-SWNT-FET a) output and b) transfer characteristics using DI water as the electrolyte.

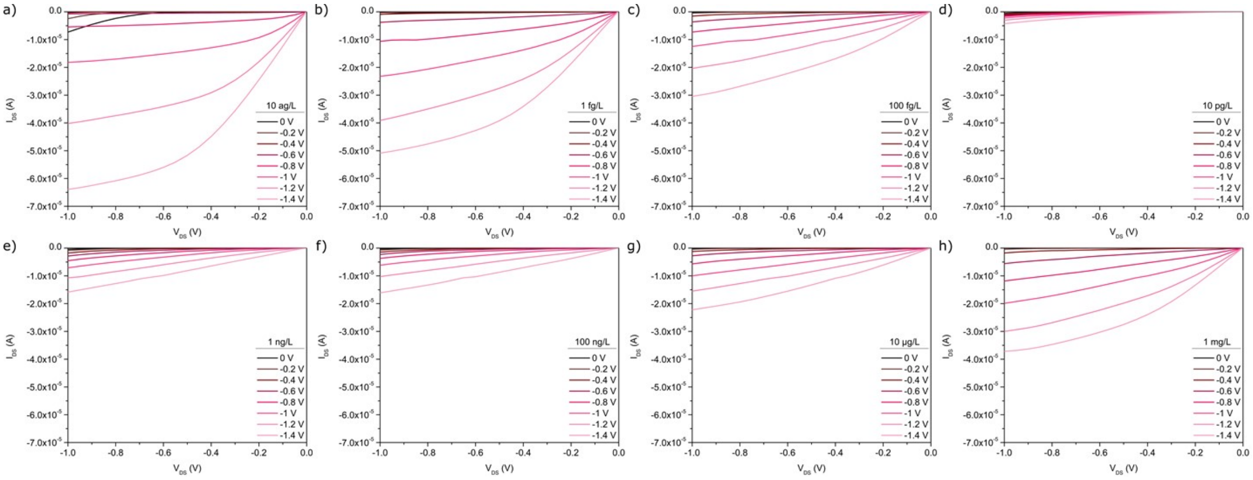

Figure S6. Output characteristics of the EG-SWNT-FET sensor using an electrolyte consisting of a) 10 ag/L, b) 1 fg/L, c) 100 fg/L, d) 10 pg/L, e) 1 ng/L, f) 100 ng/L, g) 10 µg/L, and h) 1 mg/L α-synuclein in DI water.

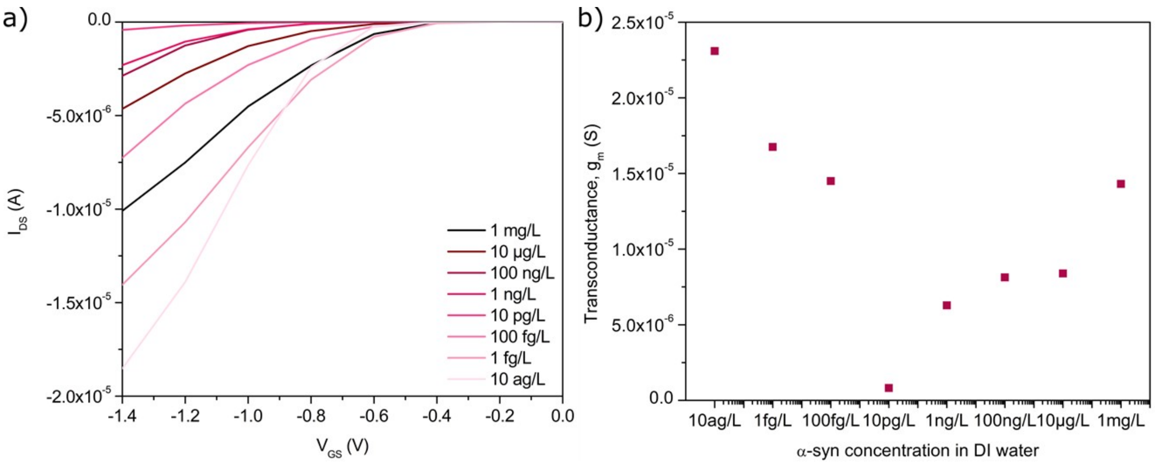

Figure S7. a) Transfer characteristics of the EG-SWNT-FET sensor using an electrolyte consisting of 10 ag/L, 1 fg/L, 100 fg/L, 10 pg/L, 1 ng/L, 100 ng/L, 10 µg/L, and 1 mg/L α-synuclein in DI water. b) Transconductance (g_m) calculated from the linear regime at all α-synuclein dilutions in DI water.
